# Correlation Index-Based Responsible-Enzyme Gene Screening (CIRES), a Novel DNA Microarray-Based Method for Enzyme Gene Involved in Glycan Biosynthesis

**DOI:** 10.1371/journal.pone.0001232

**Published:** 2007-11-28

**Authors:** Harumi Yamamoto, Hiromu Takematsu, Reiko Fujinawa, Yuko Naito, Yasushi Okuno, Gozoh Tsujimoto, Akemi Suzuki, Yasunori Kozutsumi

**Affiliations:** 1 Laboratory of Membrane Biochemistry and Biophysics, Graduate School of Biostudies, Kyoto University, Sakyo, Kyoto, Japan; 2 Department of PharmacoInformatics, Graduate School of Pharmaceutical Sciences, Kyoto University, Sakyo, Kyoto, Japan; 3 Department of Genomic Drug Discovery, Graduate School of Pharmaceutical Sciences, Kyoto University, Sakyo, Kyoto, Japan; 4 Supra-Biomolecular System Research Group, RIKEN Frontier Research System, RIKEN, Wako, Saitama, Japan; 5 Core Research for Evolutional Science and Technology (CREST), Japan Science and Technology Corporation (JST), Kawaguchi, Saitama, Japan; Universität Heidelberg, Germany

## Abstract

**Background:**

Glycan biosynthesis occurs though a multi-step process that requires a variety of enzymes ranging from glycosyltransferases to those involved in cytosolic sugar metabolism. In many cases, glycan biosynthesis follows a glycan-specific, linear pathway. As glycosyltransferases are generally regulated at the level of transcription, assessing the overall transcriptional profile for glycan biosynthesis genes seems warranted. However, a systematic approach for assessing the correlation between glycan expression and glycan-related gene expression has not been reported previously.

**Methodology:**

To facilitate genetic analysis of glycan biosynthesis, we sought to correlate the expression of genes involved in cell-surface glycan formation with the expression of the glycans, as detected by glycan-recognizing probes. We performed cross-sample comparisons of gene expression profiles using a newly developed, glycan-focused cDNA microarray. Cell-surface glycan expression profiles were obtained using flow cytometry of cells stained with plant lectins. Pearson's correlation coefficients were calculated for these profiles and were used to identify enzyme genes correlated with glycan biosynthesis.

**Conclusions:**

This method, designated correlation index-based responsible-enzyme gene screening (CIRES), successfully identified genes already known to be involved in the biosynthesis of certain glycans. Our evaluation of CIRES indicates that it is useful for identifying genes involved in the biosynthesis of glycan chains that can be probed with lectins using flow cytometry.

## Introduction

The biosynthesis of glycan chains is a multi-step process. First, free sugars are biosynthesized by sugar-specific metabolic pathways. Then, these sugar molecules are further metabolized to nucleotide sugars, which serve as donors for glycosyltransferases [1]. Specific transporters move the nucleotide sugars to the endoplasmic reticulum (ER) or Golgi apparatus [2], where they are utilized by glycosyltransferases for the tandem addition of sugars to the termini of nascent glycan chains in a sugar- and linkage-specific manner [3]. This lengthy glycosylation process requires a great number of different enzymes operating at various levels of synthesis.

Thus far, more than 300 enzymes and transporter genes have been reported to be involved in the metabolism and biosynthesis of different glycans in diverse cell types and at various stages. Each glycan structure has its own specific biosynthetic pathway. The introduction of cloning expression methodology [4], [5] has led to the successful cloning of a glycosyltransferase and to the demonstration that overexpression of a glycosyltransferase cDNA clone can confer the capability of glycan biosynthesis in overexpressing cells [6]. This mechanism is in contrast to that used by protein kinases, which also act via pathway-like processes but are often positively or negatively regulated by phosphorylation.

DNA microarray technology is very powerful because it can simultaneously detect changes in the expression levels of a large number of genes. In the field of glycobiology, extensive efforts have been made to identify the genes involved in glycan biosynthesis, and many have been shown to encode glycosyltransferases of the ER or Golgi apparatus. Many of these genes have been cloned, including those encoding large enzyme families [7]. Given the important role of gene transcription in the regulation of glycan biosynthesis, a glycan-focused cDNA microarray was developed to obtain the transcriptome of glycan-related genes [8], [9]. As the presentation of glycomic information on a cell surface is likely to be regulated at the level of transcription of the enzymes in biosynthetic pathways, a glycan-focused DNA microarray may prove useful in elucidating glycan expression [8], [10].

**Figure 1:**
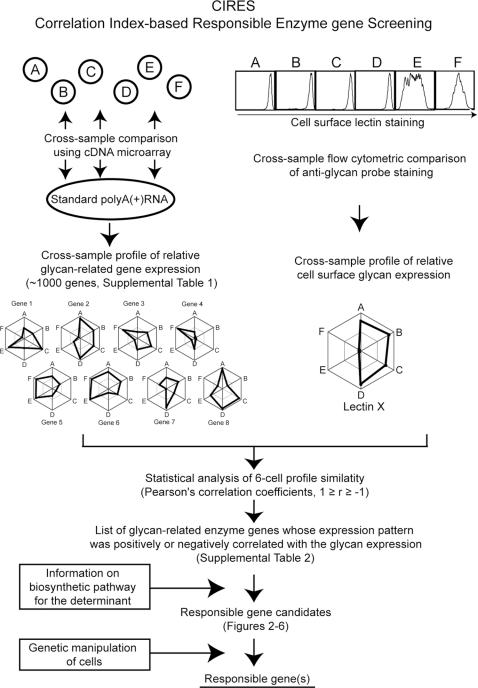
Schematic of the CIRES concept. The expression patterns of about 1000 glycan-related genes were profiled in a set of six different cell lines (A–F) by comparing the microarray binding of cellular cDNA and reference polyA(+) RNA and calculating the relative expression values (Table S1). The polygons in the left web graphs represent the relative gene expression profiles of eight glycan-related genes selected as examples. In these graphs, the difference in relative gene expression is expressed on a log scale, where the edge of the polygon corresponds to the strongest expression in each cell line (A–F). The same set of six cell lines were examined for cell-surface glycan expression using fluorescently labeled plant lectins and flow cytometry; the strength of the glycan expression is plotted as relative values among the six lines, where the edge of the polygon represents the strongest expression (web graph on top right). The glycan expression profiles were analyzed for correlations with the glycan-related gene expression profiles. Similarities and dissimilarities between the profiles were assessed using Pearson's correlation coefficient, which has values ranging from -1 (no correlation) to 1 (perfect correlation). A complete list of the genes found to be positively or negatively correlated with plant lectin staining patterns is presented in Table S2. Genes known to affect the biosynthesis of an epitope were selected from among the correlated genes (shown for each lectin in the tables on the right in Figures 2–6). A correlated gene identified by CIRES was confirmed as the gene responsible for regulating the biosynthesis of a particular glycan by transferring the gene into another cell line of the set, via gene transfer techniques such as retrovirus-mediated overexpression, and looking for a related change in epitope expression.

In the present study, we analyzed the glycan-related gene expression profiles for possible correlations with cellular glycan expression profiles in a cross-sample manner, using Pearson's correlation coefficient. This analysis successfully identified specific genes encoding regulatory enzymes for the biosynthesis of specific glycans, from among the candidate genes of the glycan biosynthesis pathways. We designated this method correlation index-based responsible-enzyme gene screening, or CIRES (Figure 1).

## Results

### Glycan-related gene-expression profiling using cDNA microarrays

The rat monoclonal antibody GL7 specifically stains germinal center B cells upon T cell-dependent antigen immunization. We recently demonstrated that GL7 recognizes the glycan Neu5Acα_2–6_-Galβ_1–4_-GlcNAc-R and that the sialyltransferase gene *ST6GAL1* is responsible for the biosynthesis of the glycan epitope recognized by GL7 [11]. By analyzing the correlation between the expression profiles for sialic acid (Sia) metabolism-related genes and the expression profiles for the GL7 epitope in a cross-sample manner, we showed that this type of correlation analysis was useful in screening for genes involved in the biosynthesis of the glycan epitope. In the present study, we further developed this systematic methodology by analyzing the correlations for cross-sample comparisons between the expression profiles for glycan-related genes and the expression profiles for various glycans, as determined by specific lectin binding (Figure 1).

The glycan-related gene expression profiles were obtained using total RNA isolated from six human B-cell lines cultured under optimal conditions, and cross-sample comparisons of these profiles were made in relation to a commercially available universal reference RNA consisting of a mixture of polyA(+) RNA from various organs. The gene expression profiles were determined as a ratio of the gene expression level to the universal reference cDNA expression level on the glycan-focused microarray ([11]; the complete relative gene expression profiles are shown in Table S1). Thus, the glycan-related gene expression profiles are expressed as the ratio of the gene expression signal at each spot on the microarray relative to the reference RNA signal.

After staining the cells with various anti-glycan probes (lectins) of known specificity, we determined the glycan expression profiles using flow cytometry. For each cell line, the transcriptional profile of glycan-related genes was used for cross-sample correlation analysis with the glycan expression profile.

### Cell-surface glycan expression profiling using flow cytometric detection of lectin staining

Cell-surface glycan expression has been extensively studied using plant lectins that recognize specific glycan epitopes. To evaluate whether correlation analyses of lectin staining and glycan-related gene expression might provide useful information, we first performed lectin staining of a set of human B cells (Daudi, KMS-12BM, KMS-12PE, Namalwa, Raji, and Ramos) to obtain their cross-sample profiles of lectin epitope expression. We analyzed the strength of the correlations using Pearson's correlation coefficient, which is a standard, well-established method for assessing correlation. To prevent possible bias in the lectin choice, we used 15 plant lectins supplied in two commercially available sets.

To evaluate the efficacy of the calculations, the lectins were first divided into two groups based on the presence or absence of previous reports asserting a correlation between cell surface expression of a specific lectin epitope and expression of a certain glycosyltransferase gene. The lectins that lacked a reported correlation were divided into highly specific (or narrow) and broadly specific groups. The highly specific (narrow) lectins were further assigned to one of two subgroups according to the position of the epitope (terminal or interior) on the glycan chain.

### CIRES correlation analyses of lectin staining profiles obtained using lectins with epitopes regulated by known biosynthetic enzyme genes

#### *Phaseolus vulgaris* leukoaggulutinin (PHA-L4)

**Figure 2:**
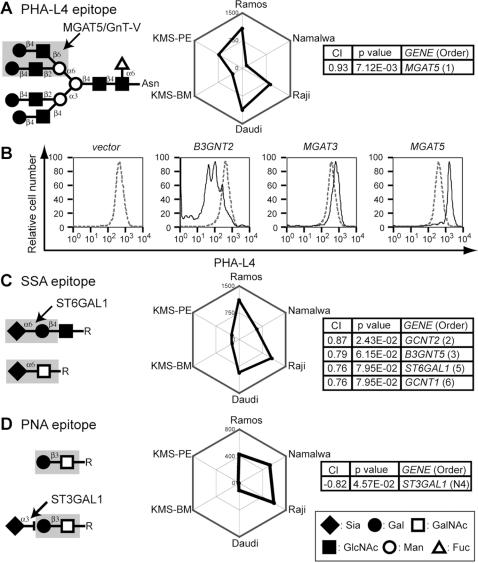
CIRES analyses of staining profiles obtained using lectins with known epitope expression-regulating enzymes. (A, C, D) Expected glycan structures for lectin recognition (left), web graphs of the lectin staining profiles (depicted as polygons) obtained using a set of six B-cell lines (middle), and the correlation indexes (CI, Pearson's correlation coefficient for profile matching) of the relevant genes that correlated with the plant lectin staining profiles and the *P* values of the correlations (right). The correlation orders of the glycan-related genes selected from the complete list of correlated genes (Table S2) are indicated as numbers in parentheses in the box for each gene, with a smaller number indicating a stronger correlation between gene expression and glycan expression profiles. Genes with a negative correlation are indicated by an N before the order number. The lectins used were (A) PHA-L4, (C) SSA, and (D) PNA. Lectin epitopes shown in the figures are taken from the literature unless otherwise specified [17], [51]. (B) Namalwa cells were infected with MSCV harboring *MGAT5-IRES-EGFP*. Control cells were infected with empty vector (*IRES-EGFP*) or the same vector encoding *B3GNT2* or *MGAT3*. Flow cytometry results for PHA-L4 staining were compared between EGFP-positive cells (solid line) and EGFP-negative cells (dashed line).

PHA-L4 recognizes tri- or tetraantennary N-glycans with β_1–6_ branching of N-acetylglucosamine (GlcNAc), which often correlates with tumor progression [12]. Histochemical and immunoblot analyses have shown that PHA-L4 epitope expression correlates with the expression of the *MGAT5* (*GnT-V*) gene [13], and this lectin is commonly used as a marker for β_1–6_-branched N-glycans. PHA-L4 epitope expression is diminished in *Mgat5*-null mice [14], and these mice exhibit enhanced rates of cytokine receptor internalization and subsequent cytokine signaling [15]. *MGAT5* expression was strongly correlated with the PHA-L4 staining profile, as shown in Figure 2A. The possible values of Pearson's correlation coefficient range between 1 and −1, where a value of 1 indicates complete correlation; therefore, the coefficient index between PHA-L4 staining and *MGAT5* expression (CI = 0.93) represents a highly significant correlation. Other correlated glycan-related genes were judged to be irrelevant to the biosynthesis of this epitope and are listed in Table S2, which contains the complete list of microarray-wide correlations for glycan-biosynthesizing genes.

The CIRES analysis correctly predicted that the *MGAT5* gene was responsible for expression of the PHA-L4 epitope. This prediction was confirmed by retrovirus-mediated gene expression in Namalwa B cells (Figure 2B). When a modified murine stem cell virus (MSCV) vector carrying genes for MGAT5 and enhanced green fluorescent protein (EGFP) divided with internal ribosomal entry site (IRES) *(MGAT5-IRES-EGFP)* was introduced into Namalwa cells, the level of PHA-L4 epitope expression was higher in the EGFP-positive population than in the EGFP-negative population. To rule out the possibility that viral infection somehow altered the cell surface glycan independently of glycosyltransferase expression, the vector carrying only *IRES-EGFP* was used as a negative control. In Namalwa cells expressing only EGFP, the EGFP-positive and EGFP-negative populations expressed identical levels of the PHA-L4 determinant (Figure 2B).

#### *Sambucus sieboldiana* aggulutinin (SSA)

SSA recognizes α_2–6_-linked Sia bound to galactose (Gal) or N-acetylgalactosamine (GalNAc). We previously showed that SSA epitope expression is induced in CHO cells by stable transfection with the rat *ST6GAL1* gene [11]. The deletion of *St6gal1* in mice eliminated the expression of the *Sambucus nigra* agglutinin (SNA) epitope [16], which is also recognized by SSA. In the present study, the correlation index was assessed to determine whether *ST6GAL1* gene expression correlated with SSA epitope expression, as determined by flow cytometry, in six B-cell lines. Although SSA staining in the six B-cell lines varied in intensity (Figure 2C), the staining profiles correlated with the gene expression profiles for *ST6GAL1* and a few other GlcNAc-transferase genes, including two β_1–6_ GlcNAc transferases and B3GNT5, which is involved in the biosynthesis of N-acetyllactosamine (LacNAc) units on glycan chains. These findings indicate that the SSA epitope detected by flow cytometry might be located at the terminus of poly-LacNAc units, which are often found on the β_1–6_ branch, and might extend beyond the glycocalyx of the cell surface.

#### *Arachis hypogaea* agglutinin (PNA)

PNA recognizes the Gal-exposed core-1 structure (Galβ_1–3_GalNAc-Thr/Ser), and the capping of this epitope by sialylation severely reduces the affinity of the interaction [17]. Diminished PNA epitope expression reportedly coincides with an increase in α_2–3_ sialyltransferase activity, which sialylates the Gal residue [18]. This change was shown to occur during thymocyte maturation, in which PNA-positive cortex cells mature into PNA-negative medulla thymocytes. In a mouse model, the deletion of *St3gal1* caused a deficiency in the derepression of PNA reactivity during thymocyte development and eventually resulted in deficient CD8^+^ T cell maturation [19].

The above findings suggest that the expression pattern of the PNA epitope might be positively affected by core-1 glycan biosynthesis and negatively affected by capping. Indeed, we found a negative correlation between PNA epitope expression and the *ST3GAL1* expression profile (Figure 2D). Thus, our correlation index analysis is able to not only identify a positive correlation but also reliably predict a negative correlation for a gene involved in the expression of a lectin glycan epitope.

Taken together, these results suggest that correlation indexing can be used to identify genes responsible for regulating cell surface expression of glycan epitopes, as determined by flow cytometry based on lectin binding. We designated this methodology as correlation index-based responsible-enzyme gene screening, or CIRES. After confirming that CIRES could be used to predict the genes involved in the biosynthesis of the glycan epitopes for these lectins (Figure 2), we used CIRES to assess the genes responsible for the staining profiles of other plant lectins, as determined by flow cytometry.

### CIRES correlation analyses of lectin staining profiles obtained using lectins that recognize specific terminal glycan structures and have unknown epitope expression-regulating enzyme genes

#### *Lens culinaris* aggulutinin (LCA)

We assessed the staining profiles of lectins that recognize terminal structures of glycan chains. LCA recognizes the biantennary N-glycan chain with core α_1–6_ linked fucose (Fuc) attached to the chitobiose [20]. The presence of a core Fuc in the N-glycan of the Fc region of IgG severely represses the antibody-dependent cellular cytotoxicity activity of the antibody [21]. The expression of *FUT8* has been shown to be responsible for the biosynthesis of a core Fuc on N-glycans [22], but a correlation between *FUT8* gene expression and cell surface LCA staining has not been demonstrated in flow cytometry experiments.

**Figure 3 pone-0001232-g003:**
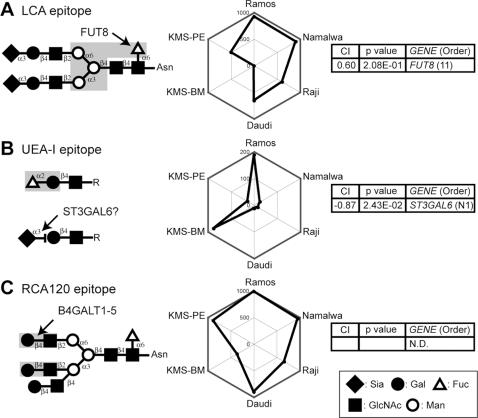
CIRES analyses of staining profiles obtained using lectins that recognize terminal glycan structures and have unknown epitope-expression-regulating enzymes. Presentation is the same as in Fig. 2 except that the plant lectins used were (A) LCA, (B) UEA-I, and (C) RCA 120. N.D. in the gene order list indicates that no gene was determined to have a correlation with the lectin staining.

Our analysis of the LCA staining profile and *FUT8* expression profile revealed a correlation (Figure 3A), although it was weaker than those for the three lectins described above (Figure 2). We also noted that *MGAT4b* gene expression negatively correlated with the LCA staining profile (Table S2). Considering that the presence of additional antennae on the N-glycan inhibits LCA binding [17], this type of negative correlation could be quite informative; however, in this case, no evidence was reported indicating that *MGAT4b* expression reduces the detection of the LCA epitope by flow cytometry. Nevertheless, CIRES is useful in predicting the genes involved positively or negatively in the biosynthesis of glycan epitopes.

#### *Ulex europaeus* agglutinin-I (UEA-I)

UEA-I recognizes α_1–2_-linked Fuc on type-2 LacNAc, which is involved in forming the epitope of H-type human red blood cell antigen [23]. UEA-I staining did not reveal a significant positive correlation with the expression of the gene for α_1–2_ fucosyltransferase, which is involved in the biosynthesis of this linkage (Figure 3B). Instead, a prominent negative correlation was found with the expression profile of *ST3GAL6*, which has a preference for type-2 LacNAc substrates on both glycoproteins and glycolipids [24].

In theory, UEA-I binding should be affected by the expression of *FUT1* or *FUT2*, as they encode the proteins responsible for H antigen biosynthesis, and by the expression of A or B transferase, which can cap the H antigen to reduce the affinity [25]. However, the sequence similarity between the A and B (and also O) transferase genes prevented their differentiation in the microarray experiments. Redundant regulation by *FUT1* and *FUT2* in these cells may be the reason that no positive correlation with UEA-I epitope expression was observed. Alternatively, these data may suggest that negatively correlated ST3GAL6, which utilizes the same substrate as fucosyltransferases, may compete with the biosynthesis of this epitope by prior sialylation of the fucosyltransferase substrate(s).

#### *Ricinus communis* agglutinin (RCA120)

RCA120 preferentially recognizes terminal LacNAc structures found in various classes of glycans. These LacNAc structures are biosynthesized by a large group of B4GalT [26] and proximal GlcNAc-transferase family enzymes. The RCA120 staining profile revealed no obvious correlation with the genes for the enzymes known to be involved in this biosynthetic pathway (Figure 3C). Given our earlier correlation results identifying a terminal glycosyltransferase as being responsible for LacNAc expression (*i.e*., sialyltransferase for SSA), this result was not surprising. It indicates that the abundant expression of LacNAc structures ensures the detection of a correlation between a terminal enzyme expression profile and the expression of a terminal glycan detected by flow cytometry. Moreover, capping of LacNAc should have a negative effect on its recognition by RCA120, which would make the detection of epitope expression more complex. It was clear that this procedure is not universally effective for lectin epitopes but that the effectiveness of the procedure depends on the type of glycan recognized by a lectin.

### CIRES correlation analyses of lectin staining profiles obtained using lectins that recognize specific internal glycan structures and have unknown epitope expression-regulating enzyme genes

#### *Datura stramonium* agglutinin (DSA)

**Figure 4 pone-0001232-g004:**
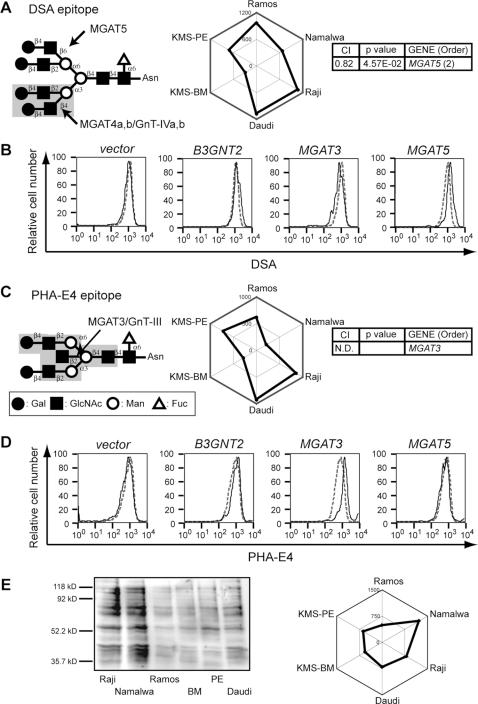
CIRES analyses of staining profiles obtained using lectins that recognize internal glycan structures and have unknown epitope-expression-regulating enzymes. Presentation is the same as in Fig. 2 except that the plant lectins used were (A, B) DSA and (C–E) PHA-E4. N.D. in the gene order list indicates that no significant correlation was detected. (B, D) Flow cytometric staining patterns for EGFP-positive Namalwa cells are shown in bold lines, and those for EGFP-negative (control) cells are shown in gray dashed lines. The overexpression of *MGAT5* resulted in a subtle (60%) increase in DSA staining. The overexpression of *MGAT3* resulted in a 2-fold increase in PHA-E4 staining. (E) PHA-E4 lectin blotting was performed using the membrane fraction of the same set of cell lines. A plot of the quantified signals reveals differences in the PHA-E4 staining profile among the six cell lines (C, E), as discussed in the text.

DSA recognizes tri- and tetraantennary N-glycans. It is specific for GlcNAc β_1–4_-Man α_1–3_-branched triantennary N-glycan [27], [28], which is biosynthesized by MGAT4a and MGAT4b. In our experiments, the DSA staining profile resembled that of PHA-L4, and thus the two lectins correlated with similar genes, most prominently *MGAT5* (Figure 4A). This result could be explained by the fact that the addition of a β_1–6_ branch increases the preference of DSA for a ligand, even though MGAT4a/b activity is required. Ihara et al. have reported that DSA staining correlates with the expression level of *MGAT5* in *in vitro*-differentiated GOTO cells [29], suggesting that *MGAT5* may also be involved in the biosynthesis of the optimal DSA epitope, with a tetraanntenary glycan. Consistent with this idea, the introduction of *MGAT5* into Namalwa cells resulted in a 60% increase in DSA staining (Mean fluorescence intensity (MFI), 1986), compared with control (MFI, 1247) (Figure 4B). Interestingly, when *MGAT3* was introduced into Namalwa cells, DSA epitope expression was subtly suppressed (MFI, 961) compared with control expression (MFI, 1222), possibly due to the competitive relationship between MGAT3 and MGAT5 [30] (Figure 4B). These effects appeared to be specific, because no obvious shift was seen in cells with introduced *B3GNT2*.

#### *Phaseolus vulgaris* erythroagglutinin (PHA-E4)

The staining profile of PHA-E4 was similar to those of PHA-L4 and DSA. This result was unexpected because PHA-E4 recognizes bisecting GlcNAc-containing biantennary N-glycans, which comprise a type of glycan distinct from the PHA-L4 epitope. Owing to the similarity among the staining patterns of these three lectins, a correlation was also found between PHA-E4 staining and *MGAT5* (Table S2), but PHA-E4 staining did not correlate with *MGAT3* (*GnT-III*), which is the GlcNAc transferase gene expected to correlate by virtue of its known epitope specificity (Figure 4C). However, when we overexpressed *MGAT3* in Namalwa cells, the MFI value of PHA-E4 staining increased, from 873 in the control population to 1868 in the EGFP-positive population (Figure 4D). Thus, the expression level of *MGAT3* appears to be important for PHA-E4 epitope biosynthesis, as expected. The overexpression of *MGAT5* had no effect on PHA-E4 binding; the MFI value was 899 in the control population and 866 in the EGFP-positive population.

When we stained the membrane fractions from the six B-cell lines using PHA-E4 in lectin-blot analyses, the blot and FACS signal strengths differed, as seen in the shape of the staining profile (Figure 4E), and *MGAT3* expression did not correlate with the signal strength on the lectin blot. Somewhat consistent with our result, Miyoshi et al. reported that *MGAT3* expression levels did not necessarily correlate with cell-surface staining of the PHA-E4 ligand in flow cytometry experiments, although co-expression was found in lectin-blot experiments [31]. Thus, our results support the suggestion of Miyoshi et al. that the cell-surface expression level of the PHA-E4 ligand epitope may be regulated by factor(s) other than *MGAT3* expression. Consistent with this idea, they also reported that the presence of bisecting GlcNAc negatively affected the sorting of glycoproteins to the cell surface [32].

### CIRES correlation analyses of lectin staining profiles obtained using lectins that recognize multiple glycan structures

Some of the lectins used in the present study had mixed or heterologous specificity. We assessed the correlation indexes for the staining profiles of these lectins.

#### *Maackia amurensis* lectin (MAM)

**Figure 5 pone-0001232-g005:**
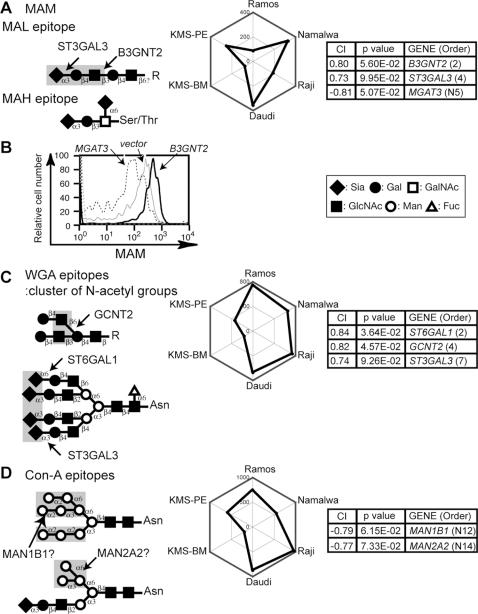
CIRES analyses of staining profiles obtained using lectins that recognize multiple glycan structures and have unknown epitope expression-regulating enzymes. Presentation is the same as in Fig. 2 except that the plant lectins used were (A–B) MAM, (C) WGA, and (D) Con-A. The epitopes of the two different lectins of MAM, MAL and MAH, are illustrated separately. WGA essentially recognizes a cluster of N-acetyl groups, as indicated, and thus required Neu5Ac as a Sia species. Con-A recognizes mannose-containing glycans with varying affinities. High-mannose-type glycans (upper diagram in (D)) bind best to this lectin. (B) Namalwa cells were infected with retroviruses encoding various GlcNAc transferases. The MAM staining patterns of the EGFP-positive populations of each infectant are shown. *MGAT5* overexpression did not shift the staining pattern in comparison with the vector control (data not shown).

MAM is a mixture of two lectin subunits, MAL and MAH. MAL binds to Sia α_2–3_-LacNAc structures [33], whereas MAH preferentially recognizes disialylated structures found in O-glycans [34]. Of the known sialyltransferases, ST3GAL3, ST3GAL4, or ST3GAL6 may synthesize the MAL epitope, and ST8s may synthesize disialylated glycans. Correlation-index analyses showed that *ST3GAL3* and *B3GNT2* may be responsible for the expression of the epitope in the six B-cell lines (Figure 5A). This is consistent with a previous report that repeating LacNAc units enhance MAL binding [35]. The MAL binding preference seemed to be more important than that of MAH in this CIRES prediction based on MAM staining and flow cytometry. As expected from its positive correlation with *B3GNT2* expression, the MAM epitope showed increased levels in Namalwa cells overexpressing *B3GNT2*, whereas overexpressed *MGAT3* was negatively correlated with the MAM staining profile, owing to the suppression of MAM epitope expression (Figure 5B). Thus, the MAM epitope may be preferentially biosynthesized on LacNAc units of the β_1–6_ branch of N-glycans. Alternatively, MGAT3 expression may change the sorting of the protein carrying the MAM epitope. Taken together, these results indicate that the expression of correlated genes can have an additive regulatory effect (positive or negative) on the cell-surface presentation of a lectin epitope.

#### *Triticum vulgaris* agglutinin, wheat germ agglutinin (WGA)

WGA is thought to preferentially recognize clustered N-acetyl groups found in N-acetylneuraminic acid (Neu5Ac), GlcNAc, and GalNAc. Neu5Ac is often a major WGA ligand because the Sia density on the termini of glycan chains tends to increase for the highly branched N-linked glycans [17]. The affinity of WGA for Sia was exploited in the isolation of Lec mutants in CHO cells [36]. The density of the N-acetyl group can also be high in the I-branched β_1–6_ GlcNAc-containing glycans [17].

The WGA staining profile correlated strongly with the expression profile of the *ST6GAL1* gene and weakly with that of the *ST3GAL3* gene (Figure 5C). (These genes were previously known as *ST6N* and *ST3N*, respectively [37].) Since WGA binding to sialylated glycans increases with the degree of sialylation, this correlation pattern seems to indicate that the supply of the substrate LacNAc is ample and that expression of the terminal sialyltransferases determines the expression level of the WGA epitope. Among these sialyltransferases, *ST6GAL1* appeared to play a more prominent role in biosynthesis in the B-cell lines used in this study. In addition to the I-branching β_1–6_ GlcNAc transferase [38], *GCNT2* expression also correlated with WGA staining, probably reflecting the GlcNAc-binding aspect of WGA. In a case such as this one, the correlation of the genes identified by CIRES may appear to be additive, but a lack of exclusivity tends to reduce the correlation index for each gene.

#### *Canavalia ensiformis* lectin (Con-A)

The modification of N-glycans occurs following the transfer of lipid-linked Glc_3_-Man_9_-GlcNAc_2_ to nascent N-glycosylated protein by oligosaccharyltransferase in the ER [39]. Con-A recognizes mannose (Man)-containing N-glycans to various degrees; it binds preferentially to high-mannose N-glycans, followed by hybrid-type N-glycans, and has the least affinity for complex-type N-glycans. In theory, oligosaccharyltransferase activity is required for the expression of the Con-A epitope, whereas mannosidase expression is inhibitory. Con-A staining profiles revealed a subtle but significant negative correlation with the expression of the mannosidase genes *MAN1B1* and *MAN2A2* (Figure 5D). Thus, the mannosidases encoded by these genes may control the expression of high-mannose N-glycans on the cell surface. Alternatively, other factors such as the expression of the protein moiety of the glycoconjugate could determine the expression of the Con-A epitope, given that the number of proteins reported to carry high-mannose glycans is limited.

#### *Agaricus bisporus* aggulutinin (ABA)

**Figure 6:**
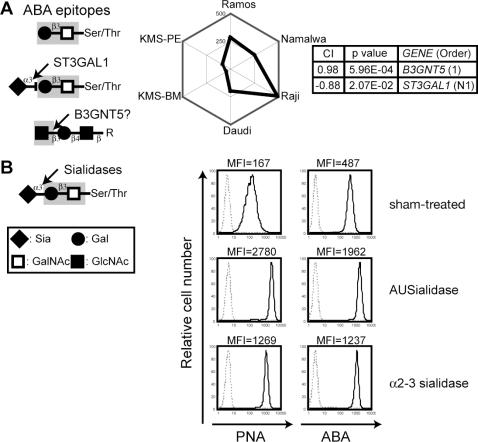
CIRES analyses of staining profiles obtained using the lectin ABA. (A) Presentation is the same as in Fig. 2 except that ABA was used. (B) Effect of sialidase treatment on the binding of ABA and PNA in B cells. (See text for the specificity of the sialidase and Fig. 2D for the PNA epitope.) Mean fluorescence intensity (MFI) values for the staining with each lectin (bold lines) are shown at the top. Dashed lines indicate the results from the non-staining control.

ABA recognizes Gal-exposed core-1 structures that are similar to the PNA epitope, but in contrast to PNA, ABA can recognize sialylated structures [40]. In B cells, ABA staining was similar to PNA staining and was negatively correlated with *ST3GAL1* expression (Figure 6A). Consistent with this negative correlation, ABA binding was increased in Daudi cells treated with sialidase (Figure 6B), similar to the enhanced binding of PNA observed upon sialidase treatment. This effect occurred with both a broad-range sialidase (*Arthrobacter ureafaciens* sialidase, AUS) and the α_2,3_-linked Sia-specific *Salmonella typhimurium* sialidase (Figure 6B), indicating that sialylation, which probably occurs on the core-1 Gal residue, somehow inhibits recognition by ABA.

*B3GNT5* expression positively correlated with ABA staining. In fact, a recent study has shown that ABA has dual specificity for glycan chains, recognizing both Gal-exposed O-glycans and GlcNAc-exposed N-glycans [41]. Whether the GlcNAc residue biosynthesized by the GlcNAc transferase is uncapped on the cell surface is unknown, but some ABA binding to GlcNAc may contribute to the increased correlation index of *B3GNT5* as compared with that for PNA, which showed an otherwise similar staining profile for the correlated genes.

### Overall findings

As confirmed by staining with various plant lectins, CIRES successfully identified enzyme genes known to be involved in the biosynthesis of lectin-binding determinants. When an unbiased set of 15 lectins was analyzed for binding to six B-cell lines, 12 of the lectins showed significant staining. Correlation assessment of these staining profiles identified the enzyme genes that are apparently responsible for the expression of the specific epitopes for nine lectins. In general, lectins that recognize terminal structures of the glycan chain tended to yield the most reliable correlation with the responsible genes.

Interestingly, CIRES also found negative correlations for some epitopes, which is consistent with the fact that CIRES results are highly dependent on the regulatory mechanisms of glycan epitope expression, some of which are negative (*i.e.*, capping of an epitope by further glycosylation). Finding negative relationships is difficult in normal biological experimental setups but may have been possible using CIRES because of the unbiased correlation coefficient calculation resulting from the large set of cross-sample comparisons.

## Discussion

### CIRES correlation analysis of glycan-related gene expression and binding of anti-glycan probes such as lectins

The functional glycans expressed on a cell surface can encode biological information. Although glycan-glycan interactions are important in determining the biological consequences of some glycosylations [42], glycan-binding proteins are the major target of functional glycans. Thus, the binding of glycan-specific proteins can be highly informative in decoding the glycomic information of an organism [43], making glycan-binding proteins a rational choice for the analysis of glycan expression. Although the identification of the proteins that bind best to each glycan is no doubt important given the role of glycan-binding proteins in glycan recognition in an organism [44], [45], here we opted to identify the genes involved in the biosynthesis of specific glycans because, as the glycan-biosynthesis enzymes are responsible for producing the glycan ligands, this information will help elucidate the regulation of biological events mediated by glycan-binding proteins.

### Systematic methodological merit of CIRES

The experimental setup for CIRES is relatively simple. Briefly, we first made a cross-sample comparison of glycan-related gene expression profiles using cDNA microarrays. This technique yielded the relative expression level for each gene as compared with a universal reference RNA, producing an expression profile for each gene. We then used anti-glycan lectin probes to obtain binding profiles in the same cell lines and compared the two types of profiles. We used the same cross-sample profiles of glycan-related gene expression for the correlation analyses of all the lectins examined in this study.

The cells that we used for gene expression profiling are available either commercially or from a non-profit cell resource center (Japanese Collection of Research Bioresources; JCRB). All cells were cultured under commonly used conditions and were used in logarithmic growth phase. Under these conditions, the glycan expression phenotype obtained by lectin staining tended to be uniform within a cell line and unique for each cell line. Thus, the data obtained by lectin staining with excess probe were suitable for determining the mean fluorescence intensity (MFI) of lectin staining.

This method compared the relative glycan expression profiles of cell lines whose cellular size and character might not be uniform; therefore, we normalized the staining signal by using the ratio of the stained sample MFI to the control MFI. This eliminates the absolute glycan expression signals and normalizes the relative expression results to the staining and data acquisition conditions.

The statistical analysis is the core of the CIRES method. Since glycosylation follows a defined pathway, we could have set up an algorithm suitable for the correlation analyses. However, we chose to use the well-established Pearson's correlation coefficient for the analyses. Regardless of the calculation method, correlations were detected even when the profiles did not completely match. Based on these calculations, a list of genes can be ordered according to the strength of their correlation. This list might be useful in designing further experiments to confirm biological functions.

In glycan biosynthesis, not all relationships are positive in nature, and some lectins used in this study yielded negative correlations. Thus, it is possible to detect dominant inhibitors of specific glycan biosynthesis steps using CIRES. Moreover, in theory, CIRES is not limited to glycan biosynthesis but could be used in any system for which numeric phenotypic expression results (such as glycan expression) can be obtained for a set of cultured cells. The correlation of non-glycosylation phenotypes with gene expression in the same system should be possible.

### Systematic methodology for using microarray data in CIRES

One limitation of the standard microarray technique is that it can only detect the relative cDNA expression levels of two samples. This was actually useful when we took the ratio of the signal for each gene relative to that for reference RNA, thus circumventing the microarray problems associated with spot-to-spot variation and hybridization variation for different nucleotide sequences. Almost all of the spots hybridized with universal reference RNA (data not shown). Normal, uniform cell culture conditions were used in this experiment to maximize reproducibility.

In order to calculate the gene expression ratios in a cross-sample manner, we wanted to avoid negative signals in the microarray scans. To do this, we directly used the raw data, instead of applying a cut-off value to the microarray signal by deducting the local background signal from the raw spot signals. We consider this to be a valid option, because we confirmed that the scanner readout after hybridization was reproducible, even for spots yielding weaker signals, and that both options yielded a similar order of relative strength (data not shown).

Normalization was also important because the array results were compared in a cross-sample manner. Sum-based normalization was applied to obtain better correlation coefficients for proven lectins (data not shown). The validity of the calculations was dependent on the use of a consistent standard, making a universal reference essential for the cross-sample comparison. In general, the microarray results tended to be more consistent for stronger hybridization signals than for weaker signals.

### Expected and unexpected findings with CIRES

For an epitope whose expression is regulated by a single gene product in its biosynthetic pathway and for which the supply of substrate for that biosynthetic reaction is abundant, CIRES will identify the gene involved in the biosynthesis of the epitope. The finding that the RCA120staining profile did not correlate with a specific glycosyltransferase was therefore not surprising, because it binds LacNAc. Generally, we obtained better correlation coefficients for epitopes created by terminal modifications of LacNAc. CIRES was also useful in identifying major genes responsible for epitope capping, which is negatively correlated with glycan expression, as in the case of PNA.

Our CIRES results tended to show correlations with the GlcNAc transferases. This may result from the use of flow cytometry to detect lectin binding, and thus glycan expression, because lectin staining preferentially detects glycans that extend beyond the glycocalyx. Moreover, the level of epitope expression may not linearly correlate with the lectin signal, because lectins tend to form multimers. As poly-LacNAcs are the usual core units for the presentation of some functional glycan epitopes, GlcNAc transferases found to correlate could also be important for the epitope presentation suitable for lectin-based recognition.

Thus far, we have not identified any enzymes other than glycosyltransferases that strongly correlate with epitope expression levels. However, a subtle negative correlation was noted between the expression of the Con-A epitope and mannosidase expression. Our failure to identify additional enzymes might have resulted from the limited number of lectins available to us. In theory, other factors involved in biosynthetic pathways, such as factors involved in sugar-nucleotide biosynthesis or transport, could also regulate glycan expression on the cell surface.

### Limitations of CIRES

Specific probes are unquestionably important for the successful application of CIRES. The most important aspect of lectin specificity is not affinity for the glycan ligand but rather the exclusivity of the enzyme. To calculate statistically significant correlations, a set of cell lines expressing different amounts of the target glycan should be used. The uniform expression of the target glycan in the cell set is undesirable.

To evaluate this methodology, we used the plant lectins that have been used extensively in other glycan studies. Since the correlations are identified by statistical calculations alone, we first assessed those lectins that had already been correlated with specific enzyme genes, to test for the identification of genes that matched glycan expression only by chance. Therefore, when CIRES is applied using uncharacterized glycan-binding probes, the biological relevance of the correlated genes must be confirmed by altering their expression in the cells. This requirement could be viewed as a limitation of the system. However, the alteration of target glycan expression has been an enduring and major objective of glycobiological experiments concerning lectins. As indicated in Figure 1, the CIRES methodology includes confirmation of the correlation by using glycan-related gene overexpression or silencing. Confirming biological relevance via the transfer of a correlated gene has the additional benefit of providing cells with a different expression level of the epitope of interest, which can be valuable for further assessment of lectin recognition in biological systems. Changing the cell surface expression of glycans for specific anti-glycan probes has been difficult, because the rate-limiting glycosyltransferase must be identified and overexpressed. Thus, allowing for a glycan-related gene transfer procedure that produces glycan-altered cells for further experiments is a major merit of the CIRES methodology.

CIRES alone cannot identify the specific glycan structure to which an anti-glycan probe binds; at the same time, knowledge of the structure of interest does not necessarily mean that modifying its expression on cells is possible. Using the CIRES method will likely result in the production of cells with modified glycan expression, but the actual structure bound by the probe may be unknown. This situation is somewhat similar to that of glycan-array binding studies, in which the probe that binds best on the glycan array does not necessarily bind the same glycan on the array as on cells and the identification of the glycan structure does not always result in the identification of the relevant biosynthetic enzyme. Thus, CIRES could be combined with conventional glycan-binding assays [44]–[46] to determine the specificity of glycan binding in a set of glycans and to identify the gene that modulates glycan expression on the cell surface, from among the pathway component enzymes. Thus, CIRES is a highly useful genetic strategy for studying the functionality of the interactions between glycans and glycan-binding proteins in cell-based systems.

## Materials and Methods

### Reagents and cell culture

Two commercially available sets of biotin-conjugated plant lectins (Plant Lectin Set I and II) were obtained from Seikagaku (Tokyo, Japan). R-Phycoerythrin-conjugated streptavidin was obtained from Caltag (USA). The B-cell lines Daudi, Namalwa, Raji, Ramos, KMS-BM, and KMS-PE were obtained from the Japanese Collection of Research Bioresources and were cultured in RPMI 1640 medium supplemented with 10% fetal bovine serum, sodium pyruvate, non-essential amino acids, and 2-mercaptoethanol.

### DNA microarrays

Gene expression profiling of the six B-cell lines was performed using a glycan-focused cDNA microarray (RIKEN human glycogene microarray, version 1) and a GEO Platform (GPL #3465) and compared with reference RNA (Clontech, USA) to create GEO Series GSE 4407, as reported elsewhere [11].

### Flow cytometry

A total of 2.5 × 10^5^ B cells in 100 µl FACS buffer (1% BSA and NaN_3_ in PBS(–)) was incubated with excess biotinylated plant lectin probes at room temperature for 30 min. R-Phycoerythrin- or FITC-conjugated streptavidin was used to detect lectin binding. Data were obtained using FACScan or FACSCaliber (BD Biosciences, USA) and analyzed using FlowJo (Tristar, USA) or Cellquest software (BD Biosciences, USA). To cross-compare staining signals between cell lines, the mean fluorescence intensity (MFI) of the background staining was adjusted to around 10, and the relative staining signal was expressed as the ratio of sample MFI divided by the control MFI.

### Statistical analysis

Fluorochrome signals on the microarray were acquired by an array scanner (Affymetrix 428) without background subtraction and were then background-corrected using a smoothing function [47]. They were then Lowess normalized using Linear Models for Microarray Data (LIMMA) [48] and the software program R [49]. Inter-array normalization was not used in cross-sample comparisons, as it seemed to cause over-normalization.

The signal from the B-cell lines was divided by the signal from the universal reference RNA [11] to obtain the relative expression profile for each gene in each cell line (Table S1). The gene expression profiles were compared with the lectin staining profiles obtained by flow cytometry. Similarities between the profiles were evaluated with Pearson's correlation coefficient, and probability values (*P*) were calculated using the correlation coefficient test. For a sample size of six, a correlation coefficient of 0.81 indicates a statistical significance level of 5%. The genes that correlated with lectin staining by this method were ranked according to correlation strength (Table S2), and this list was examined for genes that appeared to be relevant to previously reported lectin glycan epitopes and glycan biosynthetic pathways (Figure 1).

### Retrovirus-mediated transduction of glycan-related genes

Retroviruses were prepared and were used to infect Namalwa cells, as reported previously [11]. Briefly, full-length glycosyltransferase cDNA was cloned into a modified MSCV vector, which expressed cDNA and EGFP via an internal ribosome entry site. The plasmid was transiently transfected into Plat-A packaging cells [50], and retrovirus-containing supernatants were collected. Namalwa cells were spin-infected (2000 rpm, 32°C, 120 min) with the retrovirus in the presence of 6 µg/ml polybrene.

The retrovirus-infected cells were cultured for 2 days after infection before analysis by flow cytometry. EGFP-positive and -negative cells were regarded as infected and non-infected cells, respectively. The staining of these two populations was used as the control.

### Lectin blotting

B cells were sonicated in detergent-free lysis buffer [10 mM Tris-HCl (pH 7.6), 1 mM DTT, 1 mM EDTA, 0.25 M sucrose, and protease inhibitor cocktail (Roche)]. Postnuclear supernatants were further ultracentrifuged, and the pellets (membrane fraction) were resuspended in lysis buffer. The suspensions were subjected to lectin blotting with HRP-conjugated PHA-E4. The signal intensity was measured by exposure of the membrane to LAS300 (Fujifilm, Japan).

### Sialidase treatment

Sialidase treatment was carried out as reported elsewhere [11]. Briefly, Daudi cells were incubated with sialidase in 100 mM sodium acetate (pH 5.2) at room temperature prior to lectin staining. Sialidases from *Arthrobacter ureafaciens* (AUS; Calbiochem, San Diego, USA) and *Salmonella typhimurium* (Takara, Kusatsu, Japan) were used.

## Supporting Information

Table S1 Complete gene expression profiles obtained from cDNA microarray: Fluorescent Cy3 (universal reference) and Cy5 (each Bcell line) readout data were acquired from the array scanner and following statistical calculations were applied using the software program R. First, background was corrected using “Edward method”. Each data was then normalized using Linear Models for Microarray Data (LIMMA) to correct bias between fluorescent dyes. Finally, the Cy5 signal from the B cell lines was divided by the Cy3 signal to obtain the relative expression profile for each spot and resultant relative values were shown in columns I through N for each cells. Column A shows serial number of all spots on microarray and column B through E show physical location of all spots on glass slide-based cDNA microarray.
(0.31 MB XLS)

Table S2 Lists of correlated genes with lectin staining: This is an Exel file consists of 14 worksheets. First sheet shows full list of genes spotted on the Glycan-focuesed microarray and their gene ID numbers. Following sheets are the lists of genes that exhibited stronger correlation with indicated lectin staining profiles. See “Materials and Methods” for the method applied for calculation.
(0.40 MB XLS)
